# Supplementary material for: Classification of the LC4 Primarily-like Cell Line—Recapitulating a CDK4 Overexpressing Immune Evasive HIV-HCV-Induced HCC
Source: Viruses. 2025 Apr 30;17(5):653. doi: 10.3390/v17050653 (PMC12115383; doi:10.3390/v17050653)
Supplement: Supplementary file 1 [file viruses-17-00653-s001.zip › viruses-3522325-supplementary.pdf]

Figure S1

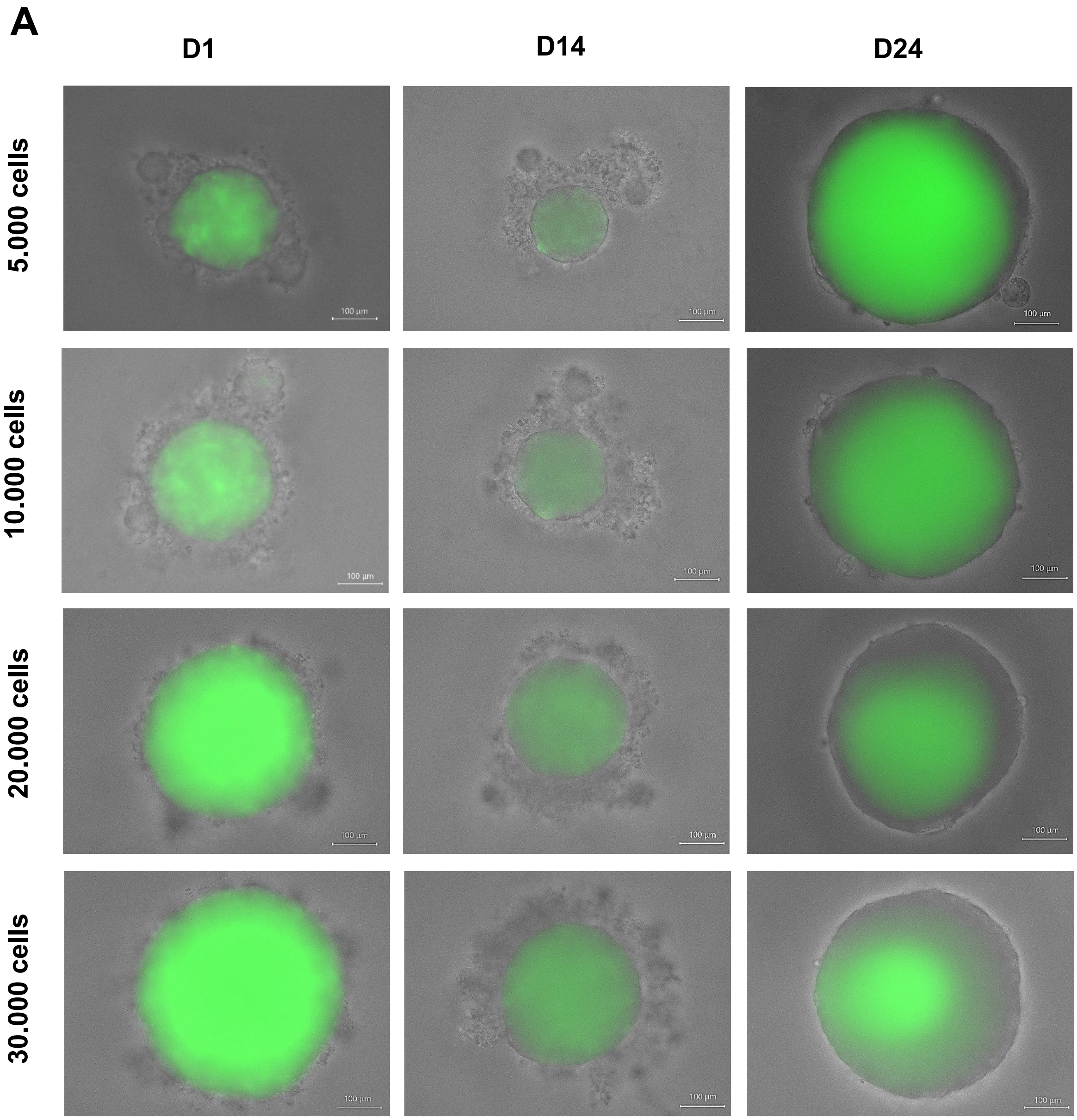
A
D1
D14
D24
5.000 cells
10.000 cells
20.000 cells
30.000 cells
100 µm

Figure S1: Representative longitudinal Observation of spheroid formation.

Figure S1 shows the time and cell number dependent formation of LC4 cell stable producing GFP. Cells were seeded on 96-well 3D culture plates with indicated cell numbers. Each well represented one formed spheroid. On indicated timepoints spheroids were captured consistently in the same wells. Visualization of the 3D spheroids was realized using the z-stack function of the fluorescence microscope BZ-9000 from Keyence (Osaka, Japan). Captures were automatically generated with the indicated magnification and consistent exposure for brightfield and GFP. Brightfield and GFP channels were merged for the visualization after preparing a full fucus of the single takes of each channel.

Figure S2

molecule	HCC core	LC4_2D	LC4_3D
ADORA2A	-1.596	-6.683	-7.314
AN1A2	-1.297	-0.923	-0.638
B2M	-0.063	-1.648	-4.126
CCND3	0.482	1.414	0.776
CCR5	-0.703	-4.451	-7.519
CD36	-1.195	-9.727	-7.912
CDKN2A	-0.73	-1.30	-2.01
CSF3R	-0.309	-8.781	-9.405
F3	-0.417	-2.082	-2.889
FGF2	-3.68	-6.609	-4.787
FLT1	-0.107	-4.596	-6.444
HMGCR	0.34	2.315	2.326
HSPA5	0.947	1.181	0.964
IKZF1	-0.035	-7.701	-8.324
IL6ST	-1.05	-2.86	-1.74
PDGFRB	-0.985	-9.65	-8.276
PTCH1	2.73	0.67	1.85
RUN13	-0.691	-5.483	-3.791
SMO	0.81	2.99	3.44

Figure S2: Detailed Heatmap of consistent Biomarker.

In Figure S2, consistently regulated biomarkers, extracted and filtered in the IPA platform were displayed in a heatmap with expression levels indicated and across the three analyzed conditions, as mentioned in the header of the heatmap. Red color indicates downregulation and blue color indicates upregulation, therefore green gene names were displayed for consistent upregulated genes, while red gene names were displayed for consistent downregulated genes, over conditions.

Figure S3

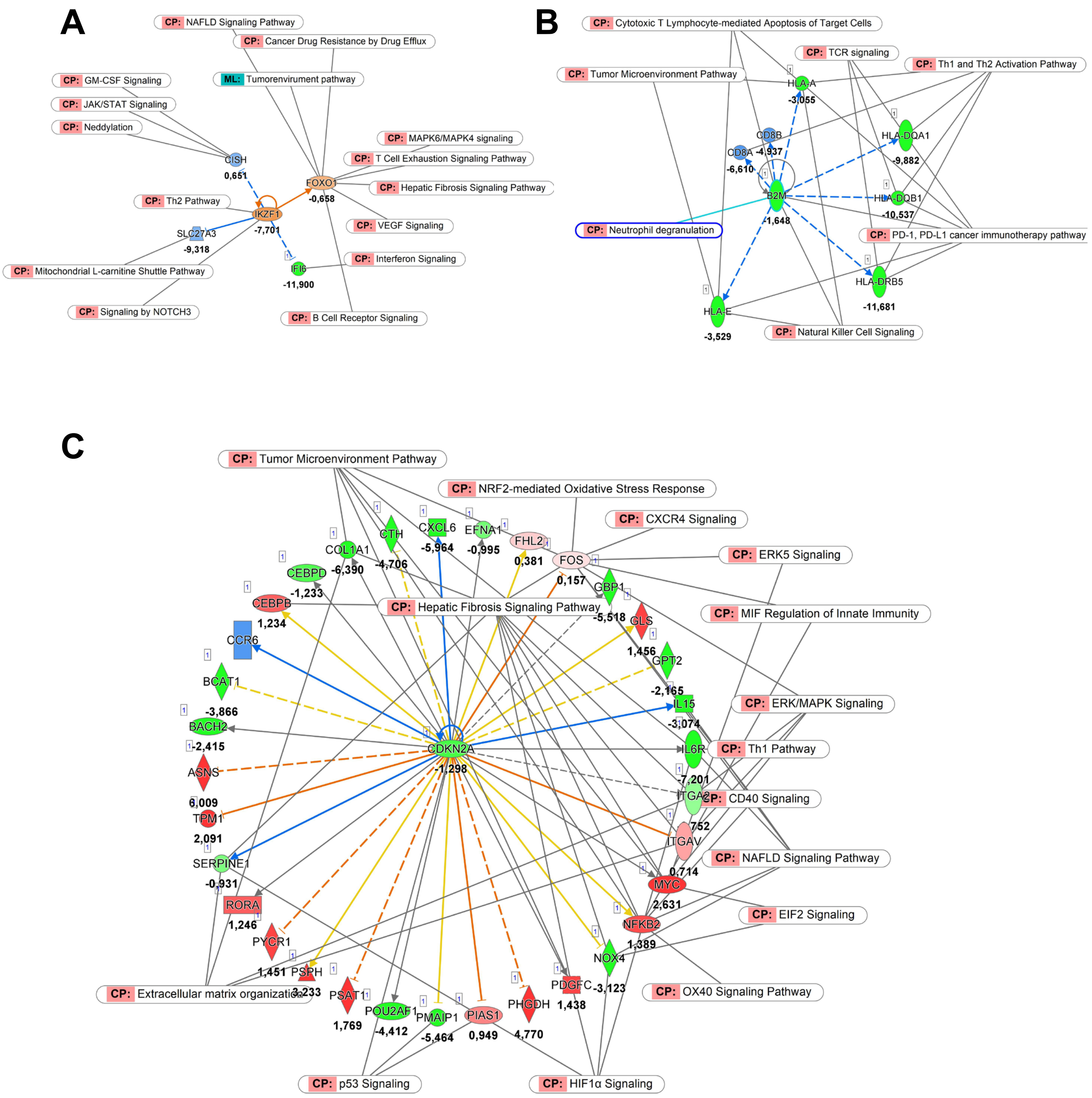
A
CP: NAFLD Signaling Pathway
CP: Cancer Drug Resistance by Drug Efflux
CP: GM-CSF Signaling
ML: Tumorenvirument pathway
CP: JAK/STAT Signaling
CP: Neddylation
CP: MAPK6/MAPK4 signaling
CP: T Cell Exhaustion Signaling Pathway
CISH
0,651
FOXO1
-0,658
CP: Hepatic Fibrosis Signaling Pathway
CP: Th2 Pathway
IKZF1
-7,701
SLC27A3
-9,318
CP: VEGF Signaling
CP: Interferon Signaling
IFI6
-11,900
CP: Mitochondrial L-carnitine Shuttle Pathway
CP: Signaling by NOTCH3
CP: B Cell Receptor Signaling
B
CP: Cytotoxic T Lymphocyte-mediated Apoptosis of Target Cells
CP: TCR signaling
CP: Th1 and Th2 Activation Pathway
CP: Tumor Microenvironment Pathway
HLA-A
-3,055
CD8B
CD8A -4,937
-6,610
HLA-DQA1
-9,882
B2M
-1,648
HLA-DQB1
-10,537
CP: Neutrophil degranulation
CP: PD-1, PD-L1 cancer immunotherapy pathway
HLA-DRB5
-11,681
HLA-E
-3,529
CP: Natural Killer Cell Signaling
C
CP: Tumor Microenvironment Pathway
CP: NRF2-mediated Oxidative Stress Response
CP: CXCR4 Signaling
CXCL6
-5,964
EFNA1
-0,995
CTH
-4,706
COL1A1
-6,390
FHL2
0,381
FOS
0,157
CP: ERK5 Signaling
CEBPD
-1,233
CEBPB
1,234
CP: Hepatic Fibrosis Signaling Pathway
GBP1
-5,518
GLS
1,456
CP: MIF Regulation of Innate Immunity
CCR6
BCAT1
-3,866
GPT2
-2,165
IL15
-3,074
CP: ERK/MAPK Signaling
BACH2
-2,415
CDKN2A
-1,298
IL6R
-7,201
CP: Th1 Pathway
ASNS
6,009
ITGA2
CP: CD40 Signaling
TPM1
2,091
752
ITGAV
0,714
CP: NAFLD Signaling Pathway
SERPINE1
-0,931
MYC
2,631
RORA
1,246
NFKB2
1,389
CP: EIF2 Signaling
PYCR1
1,451
PSPH
2,233
NOX4
-3,123
CP: Extracellular matrix organization
PSAT1
1,769
POU2AF1
-4,412
PMAIP1
-5,464
PIAS1
0,949
PHGDH
4,770
PDGFC
1,438
CP: OX40 Signaling Pathway
CP: p53 Signaling
CP: HIF1α Signaling

Figure S3: Pathways maps for consistently expressed biomarkers presented in LC4 cells with gene expression and predicate activity.

In Figure S3A, the regulatory network diagram illustrates the relationships between the consistently regulated biomarker IKZF1 and its associated canonical pathways (CP) based on IPA (Ingenuity Pathway Analysis). The graph highlights estimated inactivating (blue arrows) and activating (orange arrows) interactions of IKZF1 and downregulated genes in green derived from differential expression analysis in LC4 cells under 2D conditions. The identified pathways are involved in immune responses, cell signaling, and the tumor microenvironment, including Th1 Pathway, T Cell Exhaustion Signaling, and MAPK Signaling, emphasizing the role of IKZF1 in modulating these critical biological processes. In B the regulatory network diagram illustrates the relationships between consistently regulated biomarker B2M and its canonical pathways (CP) based on IPA analysis. The graph shows estimated inactivating interactions (blue arrows) of B2M and downregulated genes in green based on the DEG and downstream analysis in IPA of LC4 cells in 2D conditions, identifying pathways being involved in immune responses, cell signaling, and the tumor microenvironment, including TCR signaling, natural killer cell signaling, and PD-1/PD-L1 cancer immunotherapy pathways. In C the regulatory network diagram for the consistently regulated biomarker CDKN2A and its canonical pathways (CP) based on IPA analysis is shown. Upregulated genes (red), downregulated genes (green) as well as estimated inactivating (blue arrows) and activating (orange arrows) interactions of CDKN2A are displayed according to the IPA based DEG and downstream analysis in LC4 cells in 2D conditions. The network identifies pathways involved in cell cycle regulation, tumor progression, and the tumor microenvironment, such as the Tumor

Microenvironment Pathway, p53 Signaling, and Hepatic Fibrosis Signaling, underlining CDKN2A's significant impact on these pathways in liver cancer.

Table S1: antibodies for processed histological staining

antigen (clone)	supplier
anti- calnexin (C5C9)	Cell Signaling Technology
anti- CD3 (CD3-12)	BioRad
anti- CD44 (Pa5-114983)	ThermoFischer
anti- HCV Core (C7-50)	Abcam
anti- HNF4alpha (PA5-18363)	ThermoFischer
anti- ki67(OTI93)	Origene
anti- PD-L1(IHC411)	GeneTex
anti- vimentin (E-5)	Santa Cruz
anti-CD68 PG-M1)	Dako

Table S2: TaqMan assays (thermo fischer)

Gene name	Assay-ID
AADAC	Hs00153677_m1
ALB	Hs00609411_m1
CD3G	Hs00173941_m1
CD4	Hs01058407_m1
CD44	Hs01075864_m1
CD8A	Hs00233520_m1
CLRN3	Hs00380707_m1
GapDH	Hs99999905_M1
GZMB	Hs01554355_m1
HIF1A	Hs00936375_m1
IL10	Hs99999035_m1
MKi67	Hs04260396_g1
RPL0	Hs00420895_gH
RPL30	Hs00265497_m1
SLC2A1 / Glut1	Hs00892681_m1
TNFalpha	Hs99999043_m1
TP53	HS00931461_M1

Supplementary Methods

Data Processing and Differential Expression Analysis. Raw sequencing reads (n = 2 per sample) were prepared as R input data sets and further processed with R [1] and R studio as visual platform version 2024.04.1 Build 748 (RStudio 2024.04.1+748 "Chocolate Cosmos" Release (3ada7c6ddc8fcdb86a727a4f0ae467b9d9a7296c, 2024-05-07) for windowsMozilla/5.0 (Windows NT 10.0; Win64; x64) AppleWebKit/537.36 (KHTML, like Gecko) RStudio/2024.04.1+748 Chrome/120.0.6099.291 Electron/28.2.6 Safari/537.36, Quarto 1.4.553) . Input reads were trimmed for adapter sequences and low-quality bases using Trimmomatic (https://github.com/timflutre/trimmomatic) [2]. Clean reads were aligned to the human reference genome (GRCh38) using STAR aligner, (https://hbctraining.github.io/Intro-to-rnaseq-hpc-O2/lessons/03_alignment.html) version [3]. The resulting BAM files were processed with featureCounts to generate read count matrices [4]. Differential expression analysis was performed using the DESeq2 package in R version 1.42.1 (https://github.com/thelovelab/DESeq2) [5]. Genes with an adjusted p-value (Benjamini-Hochberg correction [6]) of less than 0.05 and a log2 fold change (log2FC) greater than 1 or less than -1 were considered significantly differentially expressed.

Volcano Plot Generation. A volcano plot was generated to visualize the differential expression results using the ggplot2 package version 3.5.1 (https://ggplot2.tidyverse.org/) in R [7]. The plot displays the log2 fold changes on the x-axis and the -log10 adjusted p-values on the y-axis. Genes with significant upregulation are highlighted in red, while significantly downregulated genes are shown in blue. Genes that did not meet the significance threshold are represented in gray. Genes with p-value > 200 and log fold

change <-2 and >2 were classified as top-regulated genes (top-genes) and were entirely listed in the Supplementary Table 3.

Methods for Comparison Analysis in IPA. Differentially expressed genes (DEGs) identified from DESeq2 analysis were imported into Ingenuity Pathway Analysis (IPA) software (Qiagen) version 9.1.2.16 [8]. Canonical pathway analysis identified pathways significantly enriched. The significance of the association between the dataset and the canonical pathway was measured using the ratio of mapped genes to total genes in the pathway and Fisher's exact test p-values.

Bubble Plot. Following the DEG analysis, z-scores and p-values for DEGs were extracted from IPA version 9.1.2.16 [8] with z-scores greater than 5 and p-values greater than 1.3 were visualized. The data were imported into RStudio, where p-values were transformed to -log10 values. A bubble plot was created using ggplot2 version 3.5.1 to visualize the relationship between z-scores and -log10(p-values) [7]. The size of the bubbles corresponded to statistical significance, and the color indicated z-score values. Single pathways were numbered in the graphic and listed in detail in Table 1.

Z-score based heatmaps. Data were filtered to include only canonical pathways, Biofunctions and diseases or toxicity related pathways with z-scores > 5 and p-values < 0.05. A heatmap was generated using the pheatmap package version 1.0.12 (https://cran.rstudio.com/web/packages/pheatmap/index.html) in RStudio to visualize z-scores of selected pathways [9]. Single pathways were numbered in the graphic and listed in detail in the Supplementary Table 4 and Table 2 and 3.

Correlation Heatmap. Following pathway identification in IPA, z-scores and p-values were extracted and imported into RStudio. Pearson correlation coefficients were calculated for

z-scores across samples, and the correlation matrix was visualized as a heatmap using the corrplot package version 0.94 (https://github.com/taiyun/corrplot) in RStudio. The heatmap represented the strength and direction of correlations with a color gradient for correlation coefficients [10].

Comparative Pathway Analysis Visualization in RStudio. Significant pathways related to HCC were selected from IPA, and z-scores and p-values were extracted for visualization in RStudio. Data were imported and transformed to -log10(p-values). A scatter plot was generated using ggplot2 version 3.5.1, with the x-axis representing pathways and the y-axis representing z-scores. Color differentiation indicated pathway activation and inhibition [7].

1. RCore, T., *R: A Language and Environment for Statistical Computing*. R Foundation for Statistical Computing, 2021.
2. Bolger, A.M., M. Lohse, and B. Usadel, *Trimmomatic: a flexible trimmer for Illumina sequence data*. Bioinformatics, 2014. **30**(15): p. 2114-20.
3. Dobin, A., et al., *STAR: ultrafast universal RNA-seq aligner*. Bioinformatics, 2013. **29**(1): p. 15-21.
4. Liao, Y., G.K. Smyth, and W. Shi, *featureCounts: an efficient general purpose program for assigning sequence reads to genomic features*. Bioinformatics, 2014. **30**(7): p. 923-30.
5. Love, M.I., W. Huber, and S. Anders, *Moderated estimation of fold change and dispersion for RNA-seq data with DESeq2*. Genome Biol, 2014. **15**(12): p. 550.
6. Haynes, W., *Benjamini–Hochberg Method*, in *Encyclopedia of Systems Biology*, W. Dubitzky, et al., Editors. 2013, Springer New York: New York, NY. p. 78-78.
7. Wickham, H., *ggplot2: Elegant Graphics for Data Analysis*. 2016: Springer-Verlag New York.
8. Krämer, A., et al., *Causal analysis approaches in Ingenuity Pathway Analysis*. Bioinformatics, 2014. **30**(4): p. 523-30.
9. Kolde, R. *pretty heatmaps*. 2022.
10. Wei, T. *Visualization of a Correlation Matrix*. 2024.
